# Supplementary material for: Stainless Steel as A Bi-Functional Electrocatalyst—A Top-Down Approach
Source: Materials (Basel). 2019 Jul 2;12(13):2128. doi: 10.3390/ma12132128 (PMC6651419; doi:10.3390/ma12132128)
Supplement: Supplementary file 1 [file materials-12-02128-s001.pdf]

materials

Stainless Steel as A Bi-Functional Electrocatalyst—A Top-Down Approach

Joakim Ekspong and Thomas Wågberg *

Department of Physics, Umeå University, Umeå, 90187, Sweden
* Correspondence: Thomas.wågberg@umu.se

Table 1. Data from X-ray photoelectron spectroscopy of the SSM samples showing the spectral line, binding energy (BE), Full with at half maximum (FWHM) and the atomic concentration (AC).

Line	SSM Pristine		
	BE, eV	FWHM, eV	AC, at.%
C 1s	285,0	1,25	17,73
	286,5	1,55	6,37
	288,8	1,75	3,79
O 1s	530,1	1,3	21,61
	531,3	1,5	18,29
	532,6	1,5	8,05
	533,9	1,45	1,78
Ni 2p 3/2		traces	
Fe 2p 3/2	707,0	1,3	3,01
	710,7	2,7	11,89
Cr 2p 3/2	574,1	1,2	0,92
	576,4	2,9	6,28
Mo 3d 5/2	227,8	0,85	0,12
	228,9	1,45	0,14

Line	SSM etched		
	BE, eV	FWHM, eV	AC, at.%
C 1s	284,9	1,5	8,75
	286,4	1,45	4,1
	288,6	1,35	1,89
O 1s	530,0	1,25	28,32
	532,7	1,8	23,32
Ni 2p 3/2	852,9	1,3	0,98
	855,8	2,95	1,58
Fe 2p 3/2	706,9	1,1	3,21
	710,6	3,25	15,42
Cr 3p 3/2	574,0	1,25	1,05
	576,9	1,9	10,17
Mo 3d 5/2	227,7	0,7	0,17
	232,4	1,3	1,02

Line	SSM-A		
	BE, eV	FWHM, eV	AC, at.%
C 1s	283,4	1,5	2,28
	285,0	2	13,34
	287,3	2,1	3,8
	289,0	2	3,72
O 1s	529,7	2,05	11,91
	531,3	2,35	39,05
Ni 2p 3/2	855,9	3,35	16,83
Fe 2p 3/2	711,5	3,9	9,07

Line	SSM-AR		
	BE, eV	FWHM, eV	AC, at.%
C 1s	285,0	1,35	12,64
	286,5	1,35	3,65
	288,4	1,35	3,57
	289,7	1,25	0,65
O 1s	530,1	1,45	28,72
	531,4	1,5	14,38
	532,8	1,7	3,63
Ni 2p 3/2	852,8	1,05	1,43
	854,6	2,25	8,89
Fe 2p 3/2	707,0	1,05	1,14
	710,7	2,85	16,69
Cr 2p 3/2	576,5	1,8	4,6

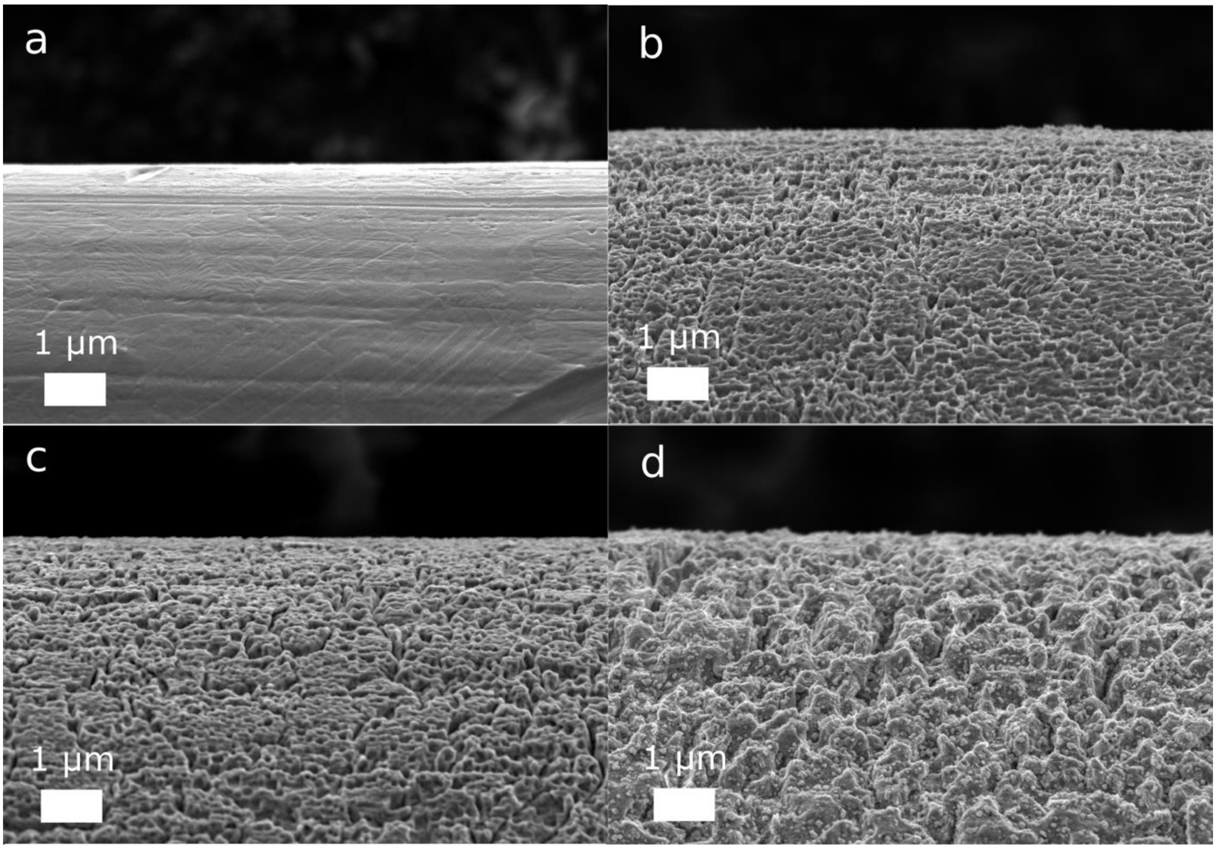

Figure S1 SEM micrographs of the stainless steel mesh (SSM) with the same magnification comparing the different stages in the synthesis procedure. (**a**) The pristine SSM; (**b**) acid etched SSM; (**c**) anodized SSM-A and (**d**) annealed SSM-AR.

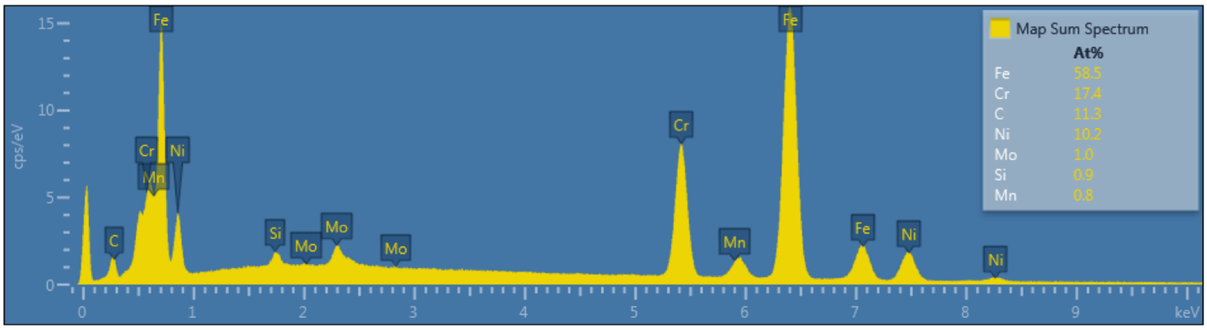

Figure S2 SEM-EDS spectrum from the pristine SSM.

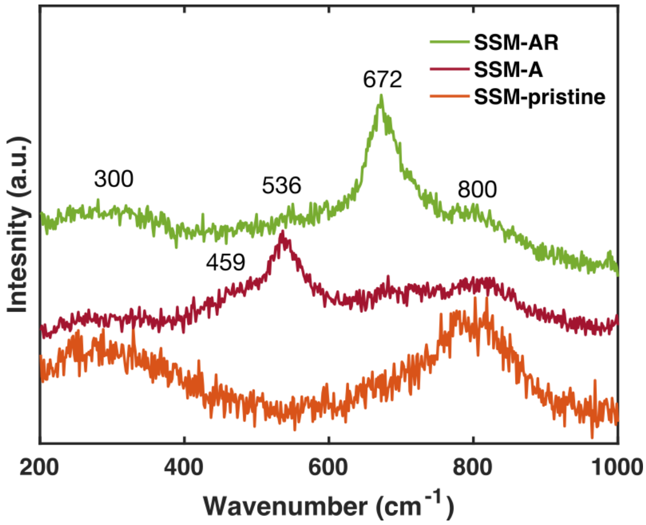

Figure S3 Normalized Raman spectra taken with an excitation wavelength of 514 nm.

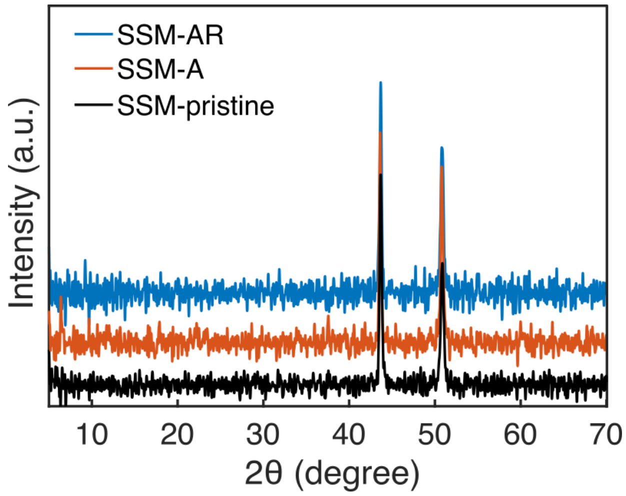

Figure S4 XRD data from the SSM samples.

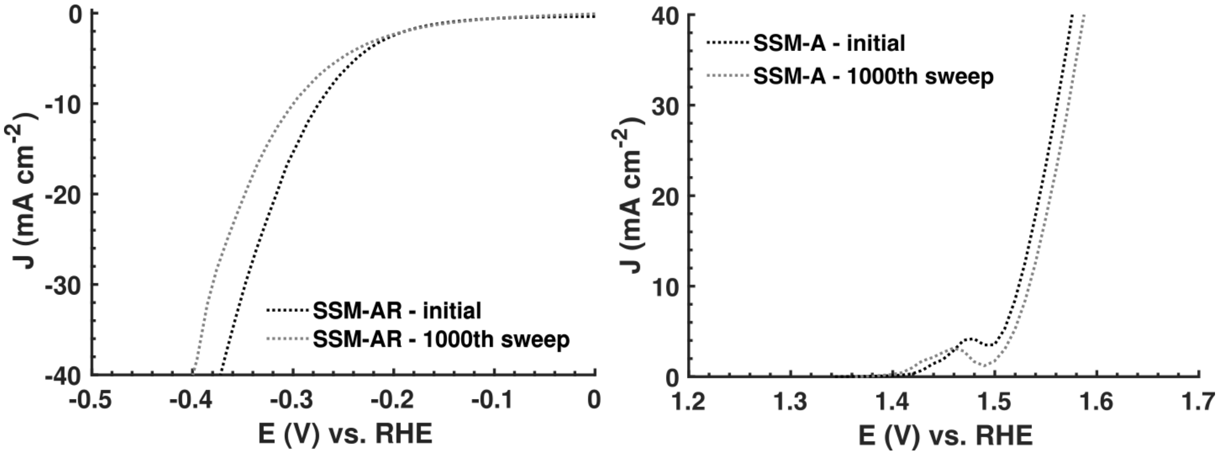

Figure S5 Electrochemical stability test of SSM-AR for HER and SSM-A for OER. The samples were measured with cyclic voltammetry for 1000 sweeps with a scan rate of 50 mVs^{-1} in 1.0 M KOH. The figure shows the initial and 1000th sweep of each electrode.
